# Multisegment transforaminal lumbar interbody fusion (TLIF) combined with Ponte osteotomy in degenerative lumbar scoliosis (DLS) surgery: a minimum of five years’ follow-up

**DOI:** 10.1007/s00264-022-05572-1

**Published:** 2022-09-24

**Authors:** Hao Qiu, Tong-wei Chu, Xiao-Jian Niu, Ying Zhang, Si-Zhen Yang, Wu-Gui Chen

**Affiliations:** 1 Department of Orthopedics, Xinqiao Hospital, Army Medical University, No. 83, Xinqiao Street, Shapingba District, Chongqing, People’s Republic of China 400037; 2 Department of Orthopedics, 907 Hospital of The Joint Logistics Team, Nanping, Fujian Province People’s Republic of China 353000; 3 Department of Spinal Surgery, Mindong Hospital, Ningde, Fujian Province People’s Republic of China 355000

**Keywords:** Degenerative lumbar scoliosis, Transforaminal lumbar interbody fusion, Ponte osteotomy, Health-related quality of life

## Abstract

**Purpose:**

To evaluate the long-term clinical outcomes of degenerative lumbar scoliosis (DLS) with the administration of multisegment transforaminal lumbar interbody fusion (TLIF) combined with Ponte osteotomy long-level fixation fusion, as well as to identify the factors affecting health-related quality of life (HRQOL).

**Methods:**

This was a retrospective single-centre study involving comprehensive clinical data. The Oswestry Disability Index (ODI), visual analog scale (VAS) outcomes, and Scoliosis Research Society (SRS-22) questionnaire were recorded to assess HRQOL. A correlation analysis was performed to determine the association between HRQOL and radiographic parameters.

**Results:**

A total of 41 consecutive patients (15 males and 26 females) met the inclusion criteria with a follow-up of 8.62 ± 1.20 years. Factors associated with HRQOL were significantly improved post-operation. Global sagittal parameters, including the sagittal vertebral axis (SVA) and T1 pelvic angle (TPA), and local parameters, including apical vertebral translation (AVT) and apical vertebral rotation (AVR), were significantly improved at the last follow-up. Significantly strong correlations between each clinical and radiographic parameter were demonstrated. Moreover, a multiple linear regression analysis demonstrated that the differences in AVT and AVR were significantly correlated with the difference in lumbar lordosis (LL), which was significantly correlated with the differences in SVA and TPA.

**Conclusion:**

The surgical treatment of DLS with multisegment TLIF accompanied by Ponte osteotomy and long-level fixations improved the quality of life of patients with a long-term effect. AVR correction is an important factor for LL restoration that significantly correlates with improvements in the sagittal balance parameters SVA and TPA, which are key factors for guaranteeing good HRQOL.

## Introduction

Degenerative lumbar scoliosis (DLS) is described as being a three-dimensional lumbar deformity that develops from the asymmetric degeneration of the facet joints and intervertebral discs [1]. DLS is often defined as de novo degenerative scoliosis with a coronal lumbar curve greater than 10° and is more prevalent in older populations [1, 2]. The updated overall prevalence of DLS has been reported to be up to 37.6%, especially in elderly females [3]. Consistent axial low back pain, disability, and neurogenic claudication are commonly reported by DLS patients with their symptoms and can lead to poor health-related quality of life. When considering the failure in adequate non-operative treatments, surgical interventions are being increasingly performed for eligible patients, and a large amount of evidence confirms the good cost-efficiency [4].

Given multiple chronic diseases, advanced age, and poor bone mass, DLS patients cannot endure major surgery. An increasing number of minimally invasive spine surgical procedures aimed at treating lumbar degenerative disease have gained public attention, such as oblique lateral interbody fusion (OLIF) and extreme lateral interbody fusion (XLIF) [5–8]. Minimally invasive surgery has been reported to have good clinical outcomes in mild-to-moderate deformities by reducing damage associated with the surgical procedure. However, the patients who are most likely to benefit have yet to be identified, and further research on the application of minimally invasive surgery approaches is urgently needed [4]. Due to the degeneration of the intervertebral disc and facet joints, DLS patients are almost consistently accompanied by stenosis, instability, and rigid deformity. Surgical treatment involves sufficient tissue release, which is not encompassed by minimally invasive surgery [9–11]. As a classic surgical approach for over two decades, transforaminal lumbar interbody fusion (TLIF) is a well-established, 3-column fusion technique for managing lumbar stenosis, instability, and deformity [12]. Furthermore, Ponte osteotomy can produce marked flexibility in extension, flexion, and rotation to guarantee adequate spine release [13, 14]. A growing number of publications have demonstrated that some radiographic parameters, such as sagittal vertebral axis (SVA), lumbar lordosis (LL), and apical vertebral rotation (AVR), are prognostic factors for degenerative scoliosis [15–17]. Two validated systems for adult spine deformity (the Scoliosis Research Society [SRS]–Schwab Classification System [18, 19] and the Roussouly classification [20, 21]) have been demonstrated to closely correlate with the health-related quality of life (HRQOL) of DLS patients.

Thus, in this study, we evaluated the long-term clinical outcomes of DLS with the administration of multisegment TLIF combined with Ponte osteotomy long-level fixation fusion and identified the factors affecting HRQOL. We further analyzed the associations among these factors to provide innovative insight into the surgical treatment of DLS.

## Materials and methods

A retrospective study was conducted to evaluate the efficacy of multisegment TLIF combined with Ponte osteotomy long-level fixation/fusion in the surgical treatment of DLS. Our work followed the Declaration of Helsinki and was approved by the ethics committee of Xinqiao Hospital of Army Medical University.

### Patient selection

The inclusion criteria were as follows: age older than 50 years; degenerative lumbar or thoracolumbar scoliosis with a Cobb angle greater than 20° on anteroposterior (AP) radiographs; back and/or leg pain refractory to conservative treatment conducted for more than one year; patients having undergone surgery between July 2011 and July 2016; and a follow-up time more than five years. Patients were grouped according to SRS-Schwab and Roussouly classifications [18, 20]. Operative improvement was defined as a decrease in sagittal modifier severity of SRS-Schwab classification or the transition from pre-operative “Mismatch” to postoperative “Match” between “theoretical” and “current” Roussouly types [22].

The exclusion criteria included adult idiopathic scoliosis, prior spinal trauma or fracture, spinal malignancy, spinal infection, and the presence of a previously implanted device.

### Surgical technique and peri-operative status

**Fig. 1:**
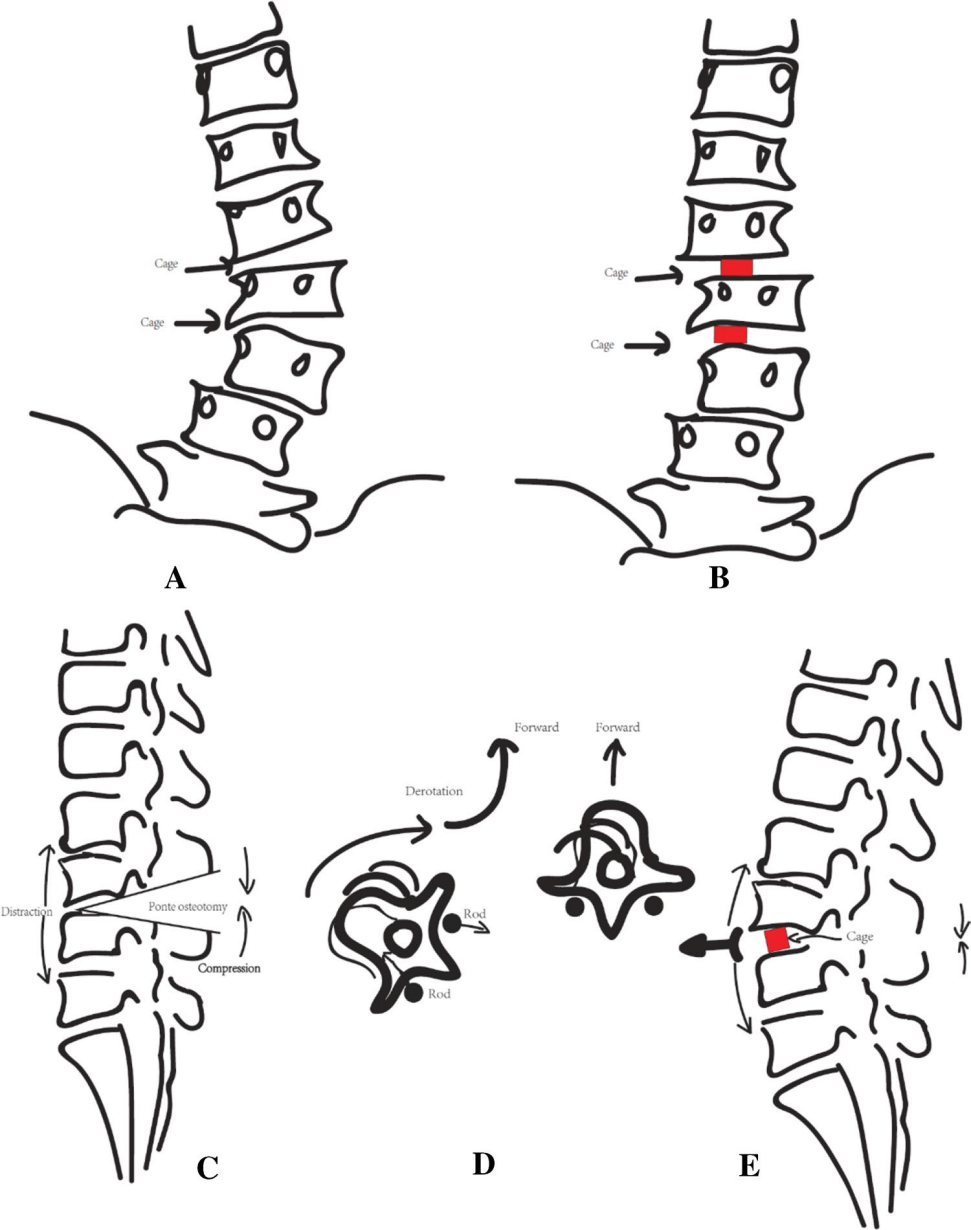
A, B An interbody cage was inserted at the narrowest site of the asymmetric disc to restore disc height. C, Ponte osteotomy was performed at the apical level of kyphosis. D, E Derotation manoeuvre was used to restore LL by moving the lumbar vertebrae forward

The patients were placed into a comfortable prone position. The lamina and articular process were revealed by using a middle incision followed by subperiosteal dissection, and pedicle screws were placed at predetermined levels. Symptomatic levels of neural compression were decompressed via the removal of the hypertrophic ligamentum flavum and facet joints. TLIF was performed at selected levels in the presence of disc herniation, lumbar stenosis, lateral listhesis or rotatory subluxation, and dynamic segmental instability. Additionally, TLIF was unilaterally performed for intervertebral release to maximize the mobility of the deformed segments. An interbody cage was inserted at the narrowest site of the asymmetric disc to restore the disc height (Fig. 1). Ponte osteotomy was performed at apical levels of kyphosis and lateral listhesis or at the rotatory subluxation segment with TLIF. The rod was contoured after decompression and the extensive release of the anterior osteophyte segments. The convex side of the rod was set first, and a derotation manoeuvre and compression were used to restore LL and to realign the spine. Subsequently, the rod derotation technique and distraction on the concave side were carefully performed to correct the lumbar scoliosis. Finally, an interlaminar bone graft was performed at all instrumented levels via the removal of the facet joint cartilage and lamina cortex.

The recorded peri-operative parameters included the operative time, intra-operative blood loss, blood transfusion amount, and the number of fused vertebrae via TLIF, instrumented vertebrae, Ponte osteotomies, and decompression levels.

### Clinical and radiographic assessments

The Oswestry Disability Index (ODI) and visual analog scale (VAS) were evaluated during the clinical assessment before surgery and at the last follow-up. Pre-operative and post-operative AP and lateral long-cassette radiographs were reviewed. Two physicians measured the films by using online Surgimap software (Version 2.2.15 Nemaris, Inc., New York, USA), and the results were recorded as averages. The coronal Cobb angle, apical vertebral translation (AVT), and coronal balance parameters, such as C7-CSVL, were measured in the coronal plane. The apical vertebral rotation (AVR) was measured by using Nash-Moe grades as a transverse plane parameter. The LL, L1-S1 length (between the midpoint of the L1 upper endplate and the S1 upper endplate in the sagittal alignment), and sagittal balance parameters, including the sagittal vertebral axis (SVA), pelvic incidence (PI), sacral slope (SS), pelvic tilt (PT), and T1 pelvic angle (TPA), were measured in the sagittal plane.

### Statistical analysis

Comparisons of the functional outcomes and radiographic parameters before surgery and at the last follow-up evaluation were performed by using paired *t* tests or Mann–Whitney U tests. The relationships between the differences in functional outcomes and radiographic parameters were evaluated by using the Spearman correlation analysis. A linear regression analysis was used to assess the factors related to the clinical and radiographic outcomes (in a stepwise manner). All of the statistical results were considered to be statistically significant if *p* < 0.05. The statistical analysis was performed with the SPSS 24.0 software package.

## Results

### Patient demographics

A total of 41 patients were included (15 men and 26 women), with a mean age of 63.0 ± 8.2 years (range: 50–78 years) and a follow-up time of 8.62 ± 1.20 years (range: approximately 5.11–10.83 years). All of the patients presented with back pain, 30 patients presented with radiculopathy, and 22 patients presented with claudication. Twenty-eight of 41 (68.3%) patients had two or more medical comorbidities, including osteoporosis (26/41), diabetes mellitus (15/41), and hypertension (14/41). To maintain the balance of the spine, the upper instrumented vertebrae were mainly located at T7-T10/T11-L2 (22/19), and the lower instrumented vertebrae were located at L5/S1 (5/16). On average, the number of TLIFs was 2.66 ± 0.69 (approximately 2–4), and the number of Ponte osteotomy procedures was 3.76 ± 0.89 (approximately 2–6). To relieve the nerve, 2.07 ± 0.82 (approximately 1–4) segments were decompressed. The mean operative time was 239.68 ± 59.44 (approximately 155–390) min, with an average blood loss of 738.75 ± 74.93 (approximately 300–2,000) ml, and the mean post-operative hospital stay was 8.38 ± 2.45 days (approximately 5–13).

### Clinical and radiographic outcomes

The distribution of patients according to Roussouly and SRS-Schwab classification is shown in Table 1. The improvement in Roussouly types was documented; specifically, 33 patients were pre-operatively classified as “Mismatch,” and 42.4% of the patients transitioned into the “Match” classification at the final follow-up. For SRS-Schwab modifiers, 43.9% of the patients were improved in SVA, 51.22% were improved in PI-LL, and 46.34% were improved in PT. The mean values of the functional outcomes, including ODI and VAS scores, were significantly improved at the last follow-up (Table 1). The ODI was 59.26 ± 9.16 before surgery and 27.91 ± 12.64 at the last follow-up. The VAS scores of low back pain were 6.29 ± 1.33 before surgery and 2.34 ± 1.24 at the last visit. Moreover, the VAS scores of leg pain were 5.41 ± 1.69 before surgery and 1.34 ± 0.88 at the last follow-up. In addition, the SRS-22 subcategories of pain, function, mental health, and total score were considerably improved after the surgery, yet the self-image domain was not significantly changed. Except for C7-CSVL, all of the radiographic parameters in Table 1 were significantly different between the pre-operative condition and the last follow-up. Global sagittal parameters, including SVA and TPA, and local parameters, including LL, AVT, and AVR (Nash-Moe grade), were significantly improved at the last follow-up. However, 11 patients(26.8%) showed distinct leg pain relief (VAS: 6.02 ± 2.43 vs 3.28 ± 1.94, *p* = 0.152), but little statistical significant improvement in quality of life (ODI score difference: 17.62 ± 8.56, *p* = 0.783; back pain VAS difference: 2.165 ± 1.892, *p* = 0.624; SRS-22 total score difference: 1.032 ± 0.816, *p* = 0.582) at the last follow-up. The long-term improvement of radiographic data was not statistically significant (SVA difference: 10.24 ± 9.24 mm, *p* = 0.846; TPA difference: 2.523 ± 1.432, *p* = 0.692; LL difference: 2.523 ± 1.432, *p* = 0.735; AVT difference: 0.923 ± 0.432, *p* = 0.592; AVR difference: 0.566 ± 0.168, *p* = 0.752).

**Table 1:** Comparison of clinical improvements and radiographic parameters between pre-operative conditions and the last follow-up

Parameter	Pre-operative	Last follow-up	Difference	t/Z	*p* value
ODI score (%)	59.26 ± 9.16	27.91 ± 12.64	31.58 ± 8.75	t = 22.901	0.000
VAS score					
Back pain	6.29 ± 1.33	2.34 ± 1.24	3.95 ± 0.45	Z = 5.550	0.000
Leg pain	5.41 ± 1.69	1.34 ± 0.88	4.05 ± 1.73	Z = 5.538	0.000
SRS-22					
Function score	1.81 ± 0.69	2.51 ± 0.42	1.63 ± 0.58	Z = 5.465	0.000
Pain score	1.92 ± 0.58	3.31 ± 0.39	1.51 ± 0.62	Z = − 5.248	0.000
Self-image score	2.21 ± 0.23	2.85 ± 0.82	0.91 ± 0.73	Z = − 0.836	0.125
Mental health score	2.63 ± 0.29	3.25 ± 0.42	1.65 ± 0.36	Z = − 3.538	0.014
Satisfaction score	0	3.41 ± 0.76	3.41 ± 0.76	0	0
SRS-22 total score	2.24 ± 0.65	3.85 ± 0.59	1.52 ± 0.26	Z = 5.634	0.000
Coronal Cobb Angle (°)	28.25° ± 14.74°	8.04 ± 6.31	21.52 ± 12.22	Z = − 5.177	0.000
C7-CSVL (mm)	14.56 ± 13.58	19.60 ± 20.13		Z = − 0.978	0.328
SVA (mm)	48.19 ± 36.04	35.71 ± 36.13	6.26 ± 48.85	Z = − 2.196	0.028
TPA (°)	21.47 ± 12.29	17.60 ± 8.48	3.87 ± 9.65	t = 2.124	0.043
LL (°)	− 31.35 ± 17.25	− 39.71 ± 14.33	8.35 ± 13.25	t = 4.05	0.000
PI(°)	46.82 ± 13.53	47.15 ± 0.25		Z = − 0.874	0.437
SS(°)	28.54 ± 14.32	32.78 ± 11.68	5.68 ± 4.76	t = − 7.82	0.000
PT(°)	19.36 ± 9.39	16.16 ± 13.25	4.66 ± 7.29	t = 5.68	0.000
PI-LL(°)	16.65 ± 11.28	8.85 ± 10.65	9.23 ± 8.59	t = 6.04	0.001
AVT (mm)	30.46 ± 17.21	15.38 ± 10.12	15.08 ± 12.22	t = 7.90	0.000
AVR (Nash-Moe grade)	2.17 ± 0.74	1.22 ± .88	1.05 ± 0.77	t = 8.69	0.000
L1-S1 length (mm)	160.86 ± 23.65	188.38 ± 23.63	22.41 ± 16.93	t = − 8.02	0.000
Roussouly classification					
Type 1	14 (34.15%)	11 (26.83%)			
Type 2	18 (43.9%)	21 (51.22%)			
Type 3 AP	4 (9.76%)	6 (14.63%)			
Type 3	3 (7.32%)	2 (4.88%)			
Type 4	2 (4.88%)	1 (2.44%)			
SRS-Schwab coronal curve					0.000
No curve	0 (0%)	0 (0%)			
Thoracic	2 (4.88%)	1 (2.44%)			
Lumbar or thoracolumbar	35 (85.37%)	38 (92.68%)			
Double	4 (9.76%)	2 (4.88%)			
SRS-Schwab PI-LL modifier					0.000
< 10°	5 (12.20%)	23 (56.10%)			
10°–20°	25 (60.98%)	10 (24.39%)			
> 20°	11 (26.83%)	8 (19.52%)			
SRS-Schwab SVA modifier					0.000
< 4 cm	12 (29.27%)	21 (51.22%)			
4–9.5 cm	19 (46.34%)	17 (41.46%)			
> 9.5 cm	10 (24.39%)	3 (7.32%)			
SRS-Schwab PT modifier					0.000
00.00 < 20°	6 (14.63%)	15 (36.59%)			
20°–30°	18 (43.90%)	19 (60.98%)			
> 30°	17 (41.46%)	7 (17.07%)			

### Post-operative complications

The incidence of early complications was 14.6%, including gastrointestinal dysfunction (4/41), superficial wound infection (1/41), and urinary tract infection (1/41). All of the complications were handled well with drug treatment. The morbidity of the late complications was 9.8%. One patient experienced screw loosening with sagittal imbalance, and one patient had an osteoporotic vertebral compression fracture. Both patients underwent revision surgery and had a good outcome during the follow-up. Two patients presented with coronal imbalance and were handled well via nonsurgical procedures.

### Correlation and regression analyses

A correlation analysis was performed for all of the clinical and radiographic parameters, as listed in Table 2. Strongly significant correlations were observed between each clinical and radiographic parameter. Specifically, the LL difference showed a strong correlations with the differences in SVA and TPA (r = 0.617 and r = 0.528, respectively), as well as correlations with the differences in AVR (Nash-Moe grade), AVT, and coronal Cobb angle (r = 0.449, r = 0.442, and r = 0.397, respectively). Further linear regression analysis was used to evaluate the parameters related to the difference in LL. The differences in AVT and AVR were significantly correlated with the difference in LL (Table 3). Additionally, the difference in LL was significantly correlated with the differences in SVA and TPA in the multiple regression analysis (Table 3).

**Table 2:** Correlation between functional outcomes and radiographic parameters

		LL diff	AVR diff	AVT diff	Cobb diff	SVA diff	TPA diff	L1-S1 diff
LL diff	*r*	1.000	0.449^**^	0.442^**^	0.397^*^	0.617^**^	0.528^**^	0.215
	*p*		0.003	0.004	0.010	0.000	0.004	0.178
AVR diff	*r*	0.449^**^	1.000	0.209	0.037	0.319^*^	0.274	0.121
	*p*	0.003		0.189	0.817	0.042	0.157	0.452
AVT diff	*r*	0.442^**^	0.209	1.000	0.647^**^	0.174	0.622^**^	0.134
	*p*	0.004	0.189		0.000	0.276	0.000	0.405
Cobb diff	*r*	0.397^*^	0.037	0.647^**^	1.000	0.249	0.337	0.314^*^
	*p*	0.010	0.817	0.000		0.116	0.080	0.046
SVA diff	*r*	0.617^**^	0.319^*^	0.174	0.249	1.000	0.524^**^	0.167
	*p*	0.000	0.042	0.276	0.116		0.004	0.296
TPA diff	*r*	0.528^**^	0.274	0.622^**^	0.337	0.524^**^	1.000	0.080
	*p*	0.004	0.157	0.000	0.080	0.004		0.684
L1-S1 diff	*r*	0.215	0.121	0.134	0.314^*^	0.167	0.080	1.000
	*p*	0.178	0.452	0.405	0.046	0.296	0.684	

^**^Correlation is significant at the 0.01 level (2-tailed)

^*^Correlation is significant at the 0.05 level (2-tailed)

**Table 3:** Linear regression analysis of the parameters related to the difference in SVA and TPA

		B	Std. error	Beta	t	*p*	95%CI
LL^a^	(Constant)	− 5.201	3.379		− 1.539	0.132	− 12.042 ~ 1.640
	AVT diff	0.433	0.142	0.399	3.040	0.004	0.145 ~ 0.721
	AVR diff	6.700	2.250	0.391	2.978	0.005	2.145 ~ 11.255
SVA^b^	(Constant)	− 15.382	6.518		− 2.360	0.023	− 28.566 ~ − 2.199
	LL diff	2.592	0.420	0.703	6.174	0.000	1.743 ~ 3.441
TPA^c^	(Constant)	0.761	1.637		0.465	0.646	− 2.604 ~ 4.126
	LL diff	0.409	0.100	0.626	4.090	0.000	0.203 ~ 0.614

^a^Dependent variable: LL diff

^b^Dependent variable: SVA diff

^c^Dependent variable: TPA diff

## Discussion

The ideal surgical treatment for DLS is a consistently important topic worthy of in-depth study and has resulted in intense discussion [2, 4]. Multiple techniques or procedures and classifications have been reported to guide the operation. Based on the presentation of DLS patients, Silva and Lenke [2] proposed a general six-tiered hierarchy for surgical treatment focused on decompression with or without instrumented fusion, with the incorporation of an anterior or posterior approach, as well as the use (or nonuse) of osteotomy. Subsequently, Schwab and colleagues [23] proposed a graduated osteotomy classification system based on six anatomic grades of resection. Due to the significant popularity of minimally invasive surgery over the past decade, Uribe et al. [24] proposed the use of six new anatomical grades of anterior column realignment, in terms of the Schwab spinal osteotomy classification. Nevertheless, excessive blood loss, tedious operation time, and complex procedures result in considerable challenges for elderly DLS patients. As a classic spinal surgical procedure, transforaminal lumbar interbody fusion is safe and easy to master with many highlights, including less blood loss, shorter operative duration, and decreased rates of hospitalization. For fractional curve correction, TLIF has been verified to surpass ALIF, as well as being observed to level the tiled vertebrae, and it also ensures good long-term outcomes [25, 26]. In this study, after the complete release of the rigid deformity, we performed TLIF by placing a large cage in the anterior sites of the disc through the local coronal concavity to level the tiled vertebra and to restore intervertebral height.

Several studies have reported a correlation between the clinical outcomes of DLS surgery and the restoration of LL. For example, Iizuka et al. [27] reported that LL values were significantly correlated with lumbar function on a back pain evaluation questionnaire. In addition, Ploumis et al. [16] reported that decreased LL (in cases of de novo DLS) is associated with poorer health status. In our study, a linear regression analysis showed that differences in the SVA and TPA were associated with the difference in LL between the pre-operative condition and the last follow-up. The restoration of LL is beneficial for SVA and TPA, which are important sagittal balance parameters. Ryan et al. [28] argued that TPA seems to show great promise in assessing patient deformities by integrating the information contained in the SVA and pelvic tilt (PT) parameters. The adequate correction of sagittal balance is related to the quality of life score reported by the patient and is beneficial for reducing post-operative complications [29]. Furthermore, Glassman et al. [30] and Koller et al. [31] reported that coronal and sagittal balance predict clinical symptoms in adult patients with spinal deformities. Therefore, we believe that the restoration of LL could improve clinical outcomes and reduce complications.

Ponte osteotomy, which involves the wide resection of facet joints, as well as of the laminae and ligamentum flavum, has been widely used to correct kyphosis by shortening the posterior spinal column and could provide flexibility in flexion, extension, and rotation for maximizing coronal, sagittal, and rotational corrections [13, 14]. Ponte osteotomy can offer a further release of deformity, which may result in overcorrections [32]. The application of this procedure has been validated to have a positive effect in adolescent idiopathic scoliosis in vertebral derotation and correction [33–35].

The SRS-Schwab system has been widely recognized as being a reliable algorithm aiding in operative planning, and modifier parameters have been shown to correlate with the postoperative HROOL of degenerative scoliosis [19, 36]. However, long-term mechanical failure, such as PJK or PJK, cannot be avoided. In contrast, based on data from asymptomatic normal individuals, the Roussouly classification could be applied to adult scoliosis patients. Through the restoration of the ideal Roussouly type, mechanical complications can be avoided [21, 37]. Therefore, the use of both classification systems for designing surgical plans may lead to optimal clinical outcomes [22, 38].

**Fig. 2:**
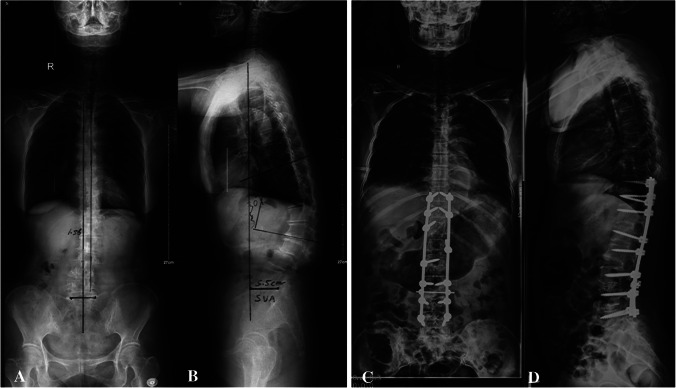
68-year-old female with degenerative lumbar scoliosis, who is receiving L3/4 and L4/5 TLIF combined with T10-L5 Ponte osteotomy long instrumented fusion. A,B: Pre-operation. PI = 39.9°, SS = 19.5°, TPA = 18.5°, AVT = 38 mm, AVR = 2.4 SVA = 55 mm; “theoretical” Roussouly-type 2; “current” Roussouly type 1; mismatch; SRS-Schwab classification: SVA = 55 mm; PT = 20.4°; PI-LL = 33.2°; ODI: 68.89%; SRS-22 Total 2.48;; C,D: 5.5 years post-operation (in the final follow-up). PI = 39.9°, SS = 30.8°, TPA = 12.5°, AVT = 12 mm, AVR = 1.3, SVA = 45 mm; “theoretical” Roussouly-type 2; “current” Roussouly type 2; match; SRS-Schwab classification: SVA = 45 mm; PT = 9.0°; PI-LL = 5.5°; ODI: 22.22%; SRS-22 Total 3.52

In this study, the selected multisegment TLIF procedures were performed at the symptomatic and spinal stenosis levels, in which multilevel Ponte osteotomy released the posterior column via inferior and superior facet joint removal, and convex facet space was compressed to restore lordosis. The combination procedure produced adequate spinal decompression and deformity corrections with less bone and blood loss and little interference with the nerves; additionally, it restored the height of the anterior column and LL. In our group, the improvements in either SRS-Schwab classification or Roussouly types were clearly evident (Fig. 2). DLS patients receiving multiple multisegment TLIF accompanied by Ponte osteotomy and long-level fixations exhibited long-term superior patient-reported outcomes with fewer medical complications. Nevertheless, 11 cases (26.8%), with poor LL correction, reported non-statistically significant betterment of HRQOL at the last follow-up. In the linear regression analysis, the factors related to the difference in LL and the differences in AVR and AVT were the two significant parameters. Ferrero et al. [39] reported that patients with larger axial intervertebral rotation had worse clinical scores. The correction of AVR and AVT were two important parameters, and the full release allowed for the lumbar vertebrae to return to the original three-dimensional position. The anterior column extension and posterior compression for LL restoration are based on AVR and AVT recoveries. In particular, when concave distraction is done without appropriate correction of vertebral rotation, the lumbar lordosis cannot be improved for lack of enough 3-column release [40]. A good anti- and mid-column release, including anterior longitudinal ligament, will get more correction of lumbar lordosis at the disc level [41]. Those patients with poor LL recovery merely underwent procedures with unilateral facet joint resection. The apical vertebrae were de-rotated inadequately owing to intact osteophytes. Moreover, improper distraction of the concavity could retard the LL correction. To achieve good outcomes, there are two key points to keep in mind. (1) A true Ponte osteotomy allows for an adequate resection of laminae and bilateral facet joints to gain sufficient flexibility of rigid degenerative lumbar scoliosis. (2) Multisegment TLIF could provide further flexibility and adequate LL with the use of large cages.

There are currently no significant consensus complication rates. Cho et al. [42] reported that the complication rate was 68% after posterior fusion, and instrumentation for degenerative lumbar scoliosis reached 68% with abundant blood loss. In the study by Charosky, the overall complication rate was 39%, with a 26% re-operation rate [43]. Moreover, Zhang et al. [44] declared that the total medical complication incidence was 25.2%, and 7.6% of the patients developed major medical complications. In our long-term follow-up, there indeed existed some complications. However, the re-operation rate was less than 10%, and the other complications were properly handled. Furthermore, the HRQOL of DLS patients improved significantly with good satisfaction.

There were several limitations to our study. We did not analyze the relationship between radiographic parameters and functional outcomes, and the risk factors for complications were not determined due to the limited sample size. Further biomechanical studies are required to confirm the stability of fusion constructs. Moreover, the relatively small sample size may have caused a selection bias, and a larger cohort is needed to address these limitations.

## Conclusion

Surgical treatment of DLS with multisegment TLIF accompanied by Ponte osteotomy and long-level fixations improved the quality of life of patients with a long-term effect. This combination treatment targeting stenosis, instability, and ridged deformity is coupled with few complications. AVR correction is an important factor for LL restoration that significantly correlates with improvements in the sagittal balance parameters SVA and TPA, which are key factors to guarantee good HRQOL.
